# Effect of acute depression associated with COVID-19 infection on health-seeking behaviour: a psychiatrist's personal account and case report

**DOI:** 10.1192/bjo.2022.521

**Published:** 2022-06-30

**Authors:** Monika Müller, Jeremy Broadhead, Thomas Simpson, Melanie A. Abas

**Affiliations:** Health Service & Population Research Department, Institute of Psychiatry, Psychology and Neuroscience, King's College London, UK; and University Hospital of Psychiatry, Bern, Switzerland; Priory Group, London, UK; University Hospital Lewisham, Lewisham and Greenwich NHS Trust, London, UK; Health Service & Population Research Department, Institute of Psychiatry, Psychology and Neuroscience, King's College London, UK

**Keywords:** Neuropsychiatry, depression, COVID-19, case report, personal account

## Abstract

**Background:**

Despite the abundant research on COVID-19-related mental health problems, little attention has been paid to acute depression occurring concurrently with the infection as a neuropsychiatric manifestation. This is important because depression is known to adversely affect help-seeking. Decreased help-seeking is likely to be aggravated by the isolation measures demanded as part of fighting the pandemic, given the disruption of social support networks.

**Aims:**

To study the effects of acute depression associated with COVID-19 infection on help-seeking behaviour.

**Method:**

We present a case report and personal account of a patient psychiatrist who developed a first onset of acute depression as part of COVID-19 infection.

**Results:**

Despite being a mental health expert the patient lacked insight into his mood change and its negative effect on help-seeking behaviour, resulting in reliance on a family caregiver to raise the alarm.

**Conclusions:**

For those experiencing this complex interaction between COVID-19 infection and the brain, social support will be needed to ensure timely presentation to the healthcare system. Greater attention to behavioural change as part of COVID-19 infection is needed to optimise treatment outcome.

Although confusion and delirium have been identified as the most frequent neuropsychiatric manifestations of COVID-19, a variety of acute psychiatric manifestations, including depression, have been reported.^1–6^ According to an analysis of 62 000 people without history of mental illness and diagnosed with COVID-19 in the USA, 22.5% had neuropsychiatric manifestations^4^ and 6% were diagnosed with a newly developed psychiatric illness.^5^ Similar findings are reported from other countries.^2,3^ Individuals with COVID-19 symptoms are almost three times more likely to have clinical depression than those with asymptomatic COVID-19.^6^

Most research on depression and COVID-19 has been conducted retrospectively, with relatively little attention to acute mood disorders occurring concurrently with the infection. This is important not only because of the added impact on quality of life, but because depression is known to adversely affect help-seeking. One meta-analysis found that people with depression are three times more likely than those without depression to be non-adherent to recommendations regarding any type of medical treatment.^7^

Potential explanations include negative cognitions, anhedonia and hopelessness. In line with Beck's cognitive triad,^8,9^ the more severe the symptoms of depression, the more negatively biased is the individual against seeing benefit from professional support.^10,11^ Previous studies showed that social support increases help-seeking of individuals with depression.^12–14^ Social support may be especially reduced during the pandemic as a result of the containment measures to control the spread of the infection. Here we present a case of a consultant psychiatrist who developed a first onset of a mood disorder as part of COVID-19 infection, which was primarily characterised by negative cognitions and which interfered with help-seeking. He relied on his partner, also a consultant psychiatrist, to attain medical care. Both give their personal account of navigating through the healthcare system:

‘We want to share our experience as patient psychiatrist and caregiver psychiatrist because it was disturbing to realise that somebody without a psychiatric history can suddenly develop severe psychiatric symptoms that interfered with help-seeking to the extent that COVID-19 became life-threatening. Our experience showed us how tiring a patient journey within the healthcare system can be and how deleterious this can be for people whose motivation to seek medical help is markedly reduced as a result of their mental health problem.’ (J.B. and M.A.A.)

## Method

The patient (J.B.) and his partner (M.A.A.) are both co-authors of this paper and give their personal account as patient psychiatrist and family caregiver psychiatrist alongside the objective description of the case report. Written informed consent was obtained from the patient retrospectively after full recovery. Ethical approval was not required for this study because it is a first-person account.

## Results

### Case presentation and personal account

On 26 April 2020, during the first lockdown in the UK, a 61-year-old consultant psychiatrist developed cough and fever. COVID-19 was confirmed by PCR testing on 27 April 2020. In accordance with UK governmental containment measures he called NHS 111 (the National Health Service's non-urgent medical helpline) for medical advice. This telephone system was remotely triaging people with possible COVID-19. The service proposed symptomatic treatment with paracetamol and self-isolation at home with his partner. Despite this measure, symptoms worsened with persistently high fever, cough, fatigue, severe muscle pain, loss of appetite and poor sleep:

‘At the beginning I felt ill but was not worried. I was confident I would recover, although medical check-up would have been reassuring when I was worse after one week. I called NHS 111 again at day 8.’ (J.B.)

They advised the patient to continue with paracetamol and self-isolation. At the suggestion of a medical colleague, the patient bought a pulse oximeter. His oxygen saturation on day 9 was about 92% and his temperature persisted at 38.5°C. The situation worsening, the patient and his partner decided not to call NHS 111 for a third time, but to attend an accident and emergency department. He was seen by a triage doctor but sent home because oxygen saturations at the time of the visit were ≥94% sustained on ambulation, which did not prompt further investigation. Physical and then mental state continued to worsen. The patient looked and felt very unwell, became inactive, slept a great deal and became morbidly negative. He lost all interest and motivation to read, listen to the radio or watch television:

‘I could not be bothered to make a cup of tea and if my partner had not insisted I would not have eaten or drunk. I began to think my life had been pointless, in particular that I had done nothing useful and had never achieved anything to be proud of. With this idea, that my life was a failure, I could not see possibility for any fulfilment in the future. I felt nothing would change for the better. The future would be more of the same and futile. I can say that I became hopeless and indifferent, with the whole life enterprise seeming desperate. I did not feel particularly sad, but this way of thinking and a complete numbness for existence took over. I was not suicidal but I wasn't concerned to die.’ (J.B.)

On day 10, the patient decided he wanted to sell the house. The property had been much enjoyed and he had been renovating it for 4 years. He was convincing, telling his partner that with the last renovations he had spoiled it, its charm was lost and he could not bear to stay there. In a phone call with his daughter he told her he wanted to get rid of the family dog, which he believed was ‘in some way evil’. This was a family dog he was fond of and had owned for 12 years:

‘Usually I am very involved in the garden and house, but in the state I was, I was hopelessly disappointed in it all and wanted to move. It did not occur that this wish might be temporary. An additional preoccupation was about possibly having mismanaged a patient as a very junior doctor and the need to report it to the police.’ (J.B.)

It was not possible to change these ideas through gentle argument. On the contrary, the patient persisted in wanting to leave the house, this time blaming changes made in the garden. His family did not challenge but deferred the need to make decisions:

‘Initially, his negative views and statements about selling the house affected me because I took them at face value, thinking he really meant them and perhaps he just had not told me before. It was only when my daughter told me that he had contacted her to say he thought we should sell the dog as he didn't like it anymore that I started to realise that his thinking was unusual.’ (M.A.A.)

The patient was withdrawn and would sit isolated and uncommunicative. He remained fully oriented for place, time and person and his partner did not observe fluctuations in attention or concentration, or increased irritability, which might have suggested delirium:

‘I completely withdrew. To seek medical help was a struggle and probably purposeless as I was convinced that I would again be sent home. I was sure that nobody was able to help and thought negatively about the front-line healthcare professionals. The idea that they did not really want to help was dominant. Also I thought I may be making excessive fuss and should not be wasting the time of the staff at a busy hospital. I was completely dependent on my partner to take the lead and take steps to engage with the healthcare system.’ (J.B.)

In this situation his partner began to worry and insisted on medical help, driving the patient on day 12 to the accident and emergency department for a second time:

‘After about 10 days of COVID-19 he was tending to just sit in a chair all day, often in a separate room from where I was. I had to take more of an active role, speaking to medical colleagues, taking him to hospital and take decisions on his behalf in order to make sure that he got the right care.’ (M.A.A.)

He was admitted on the 7 May 2020 to a COVID-19 respiratory ward and diagnosed with COVID-19 pneumonitis (leucocytes 6.6 G/L, ref.: 3.7–9.5; CRP 76 mg/L, ref.: <5 mg/L; ferritin 2786 μg/L, ref.: 24–336 μg/L). We have retrospectively calculated his International Severe Acute Respiratory and Emerging Infection Consortium (ISARIC 4C) scores.^15,16^ The patient's ISARIC 4C mortality score was 8/21, corresponding to a 14% risk of death, and his ISARIC 4C deterioration score was 420, which corresponded to 37% risk of deterioration. The patient's age, gender, respiratory frequency and laboratory results explained the scores. He had no history of physical or mental illness. He was treated with high-flow oxygen through a Venturi mask, anticoagulants, intravenous antibiotics and 40 mg prednisolone daily. On discharge 10 days later his mental status had normalised:

‘What preoccupies me particularly is that I am a medical doctor and yet I was unable to seek help because of my negativity. The change was sudden. I did not have a psychiatric history. This suggests that anybody could get into serious difficulties if not well supported. If I had lived alone and if my partner, herself a psychiatrist, was not insisting on seeking medical care, the COVID-19 could have become life-threatening for me. I wonder, how many affected persons with weak social support aggravated by lockdown isolation might have seriously deteriorated or even died at home.’ (J.B.)

‘Retrospectively, this experience showed me the responsibility informal caregivers may have to take to ensure family members living with mental illnesses get appropriate medical care. I also realised that the family is key when setting up a physical healthcare plan for those with comorbid mental disorders.’ (M.A.A.)

## Discussion

This combined case report and personal account provides insights about an acute neuropsychiatric manifestation of COVID-19, which was characterised by depressive symptoms and interfered with help-seeking. The neuropsychiatric symptoms included negative thinking, with rumination, anhedonia, lack of motivation and interest, loss of hope, social withdrawal, poor sleep, fatigue and loss of appetite. One might argue that these symptoms are an expression of the underlying infection rather than of depression and thus question the neuropsychiatric diagnosis. We think that especially the pathological thinking pattern, the loss of hope and the social withdrawal cannot be explained merely by a COVID-19 infection. Other arguments underlying this hypothesis are the neurotropic potential of COVID-19,^1–6,17^ the fact that the patient prior to the infection had no history of mental illness, and the close link between the course of the neuropsychiatric disorder and the COVID-19 infection. It had an acute onset about 2 weeks after the patient tested positive and remitted completely without the need for psychotropic medication once COVID-19 was treated successfully. One differential diagnostic alternative to a mood disorder would be delirium, the most common neuropsychiatric presentation of COVID-19.^1–6^ The patient's partner, a consultant psychiatrist herself, was unable to detect symptoms that might have suggested delirium. The patient remained fully oriented for place, time and person and neither fluctuations in attention or concentration nor increased irritability were observed. We therefore submit that the diagnostic criteria for ‘mood disorder due to known physiological condition with major depressive-like episode’ are fulfilled.

In this case, the pathological thinking pattern merits special attention as it was characterised by the cognitive triad, a typical feature of depression, and was at the core of the patient's reduced help-seeking behaviour.^8,9^ The patient had thoughts of guilt and failure (‘My life had been pointless […] I had done nothing useful and had never achieved anything to be proud of’; ‘I had mismanaged a patient [and] need to report it to the police’; ‘I may be making excessive fuss and should not be wasting the time of the staff at a busy hospital’); a negative view of others (‘I was sure that nobody was able to help and thought negatively about the front-line healthcare professionals [because] they did not really want to help’); and a negative conception of the future (‘I could not see possibility for any fulfilment in the future. I felt nothing would change for the better’; ‘I became hopeless and indifferent, with the whole life enterprise seeming desperate’). This illustrative example reminds us that low mood is only one of several symptoms of depression and that a pathological thinking pattern can lie at the core of its clinical presentation.

This may warrant consideration not only when diagnosing patients with depression but also when planning psychotherapeutic interventions specifically targeting negative cognitions, such as cognitive restructuring.

### Strengths and limitations

Limitations of this study include a lack of information about the central nervous system infection and about the severity of the depression using a validated scale. Routine clinical management of COVID-19 patients within the NHS does not include a lumbar puncture or cerebral magnetic resonance imaging, nor an evaluation by a mental health professional. As this article was conceived after the patient's full recovery it was not possible to complete these diagnostic evaluations *post hoc*. However, we consider the integration of the description of COVID-19-related acute depression and the lived experience of the patient and his partner, both consultant psychiatrists, a major strength. The behavioural changes resulting from this neuropsychiatric manifestation of COVID-19 demonstrate the importance of ensuring physical healthcare for those with mental disorders. We cannot leave potential life-saving medical care solely dependent on a patient's motivation, which is, as seen in this illustrative example, all too often hampered by the mental illness itself. Despite the patient being a consultant psychiatrist he was unable to realise the changes in his mental status leading to reduced help-seeking behaviour and placing him at risk for a life-threatening course of COVID-19 infection. Detection of altered motivation and behavioural changes as expression of an underlying mental health problem, using techniques to enhance motivation, and the integration of significant others into therapy should lie at the core of our efforts as mental health professionals.

### Help-seeking behaviour and service response in COVID-19-related depression

Individuals with depression often show reduced help-seeking behaviour^7,18–20^ and evidence suggests that social support increases it.^12–14^ The problem of decreased help-seeking in this vulnerable population is likely to be compounded by coronavirus containment measures, which typically disrupt social support networks. Social distancing and restriction of social interaction slow the spread of COVID-19 by ‘flattening the curve’^21–23^ and thus decreasing mortality.^24–26^ But they may also increase loneliness and reduce social support.^27,28^ Social isolation is deleterious to health in general and the magnitude of risk associated with it is comparable with that of cigarette smoking and other major biomedical and psychosocial risk factors.^29,30^ One postulated mechanism of action is the influence of significant others on health-seeking behaviour.^31^ Evidence indicates that early treatment of COVID-19 is likely to reduce mortality.^32,33^ This suggests that late presentation to the healthcare service because of reduced help-seeking behaviour places patients at risk of poorer outcome.

A further consideration in the UK health system as part of the COVID-19 response is that patients seeking medical help are triaged by phone. Those who are not considered in need of urgent medical attention are asked to ‘call back in 2 days’ if still feeling ill, which relies on the patient being sufficiently motivated. There is no specific protocol to evaluate major depression or any other neuropsychiatric disorder likely to influence motivation. We argue that there is need for more attention to behavioural change as part of COVID-19 infection. Emergency services screening patients at home should briefly evaluate the patients’ motivation to call back. Those with low motivation, for example as a result of concurrent depression, might be flagged and proactively followed up. Additionally, it might be desirable to include the Patient Health Questionnaire 2 (PHQ-2), an ultra-brief tool consisting of two items to screen for major depression.^34^

We are now seeing a shift in the dynamic of the pandemic allowing us to move from emergency planning to long-term management of COVID-19 at a population level. Instead of confinement measures affecting all citizens in the same manner, we advocate a more targeted approach taking into account the complex needs of vulnerable populations, such as patients with mental illnesses in general and individuals suffering from depression in particular. We should consider the importance of social support for these patients as their help-seeking behaviour is typically reduced as part of their illness. Importantly, this case report and personal account also shows that acute depression can be a neuropsychiatric presentation of COVID-19 itself, placing all of us potentially at risk for this complex interaction between COVID-19 infection and the necessity of social support to present to the healthcare system in time to have the best chance for optimal treatment outcome.

We conclude with the words of J.B. and M.A.A.:

‘Our experience as patient psychiatrist and caregiver psychiatrist taught us how important it is to really engage depressed patients in order to ensure access to care. As the patient I have now experienced the same ambivalence about accepting help from professionals that my patients have sometimes expressed towards me. As the caregiver I needed to carefully weigh the extent of getting involved in the care of my partner. On one hand I did not want to impose myself on his treatment decisions and I did not want to be perceived as a demanding medical relative by the healthcare professionals. On the other hand, I realised I had to take a more active role on his behalf to make sure that he got the right care. We both realised the key role that informal caregivers play in the setting up of an adequate care plan and the importance of taking the concerns of significant others into account – regardless of how unimportant their queries seem to be in our eyes.’

## Data availability

The authors confirm that the data supporting the findings of this study are available within the article.
